# Concussion is confusing us all

**DOI:** 10.1136/practneurol-2015-001087

**Published:** 2015-06

**Authors:** David J Sharp, Peter O Jenkins

**Affiliations:** Computational, Cognitive, and Clinical Neuroimaging Laboratory, Division of Brain Sciences, Faculty of Medicine, Imperial College London, Hammersmith Hospital Campus, London, UK

**Keywords:** Concussion, Post-concussive, Traumatic brain injury, Mild traumatic brain injury, TBI

## Abstract

It is time to stop using the term concussion as it has no clear definition and no pathological meaning. This confusion is increasingly problematic as the management of ‘concussed’ individuals is a pressing concern. Historically, it has been used to describe patients briefly disabled following a head injury, with the assumption that this was due to a transient disorder of brain function without long-term sequelae. However, the symptoms of concussion are highly variable in duration, and can persist for many years with no reliable early predictors of outcome. Using vague terminology for post-traumatic problems leads to misconceptions and biases in the diagnostic process, producing uninterpretable science, poor clinical guidelines and confused policy. We propose that the term concussion should be avoided. Instead neurologists and other healthcare professionals should classify the severity of traumatic brain injury and then attempt to precisely diagnose the underlying cause of post-traumatic symptoms.

## Introduction

As neurologists, we often see patients who have persistent neurological problems after head injuries. Many of us are happy to reassure them that they have had a concussion and are suffering from transient ‘postconcussion syndrome’. These labels provide reassurance, both to the neurologist and patient, that the injury is benign and reinforce the view that nothing can be done to help. But what does concussion mean, and is such therapeutic nihilism justified? Although a ‘light touch’ to mild traumatic brain injury (TBI) is often appropriate, many patients go on to have persistent problems that would benefit from more precise neurological assessment.

TBI is a common problem. There are estimated to be at least 1 million emergency department attendances each year in the UK due to head injuries, 90% of which have been considered to be mild.^1^ Mild TBI is often considered relatively harmless. The assumption is that any neurological dysfunction is short-lived, usually in the region of minutes. However, long-term effects can be surprisingly common. The resolution of obvious confusion is often followed by a constellation of symptoms that include headache, dizziness, fatigue, irritability, reduced concentration, sleep disturbance, memory impairment, anxiety, sensitivity to noise and light, blurred vision and depression. Most patients suffering a mild TBI recover in the first 3 months,^2–4^ but a significant minority (up to a third) report symptoms persisting beyond 6 months.^5–7^ The presence of a more severe initial injury, pre-existing psychological problems, older age, female sex and previous head injuries all increase the likelihood of persistent symptoms.^8^ In addition, involvement in a compensation claim can also be a significant factor in perpetuating symptoms.^9^ ^10^

TBI can also lead to long-term effects including epilepsy and neurodegeneration. There is an increased risk of Alzheimer's disease, Parkinson's disease and chronic traumatic encephalopathy.^11–13^ Since the early 20th century, repetitive brain trauma sustained from boxing was recognised to produce a progressive neurological deterioration. Originally termed ‘dementia pugilistica’, there has recently been renewed interest in what is now termed chronic traumatic encephalopathy, a condition defined by neuropathological findings including the presence of neurofibrillary tangles in the depths of sulci.^14^ Epidemiological studies also show increased mortality rates even after mild TBI. One large cohort study tracked patients with TBI of all severities attending emergency departments in Glasgow, UK, in 1995 and 1996.^15^ ^16^ Thirteen years after injury the mortality rate of the group had reached over 40%, with increased mortality even in young patients after mild TBI (∼15 vs 2 per 1000 per year in community controls).^15^ This did not simply reflect non-specific lifestyle factors associated with those exposing themselves to likely injury, as patients with mild TBI had higher mortality rates than those with other types of injury.^16^

There are obviously important questions to answer about the way mild TBI is managed and the extent that patients need to be followed-up. There is confusion about acute assessment and treatment, as well as uncertainty about the prevalence of neurodegenerative complications and the ‘dose’ of TBI needed to produce them. We need clinical research to answer these questions, and clear guidelines about the acute management of mild TBI. Both of these goals are hampered by the confusion that surrounds the use of the term concussion.

## Concussion through history

Concussion has been applied in numerous different and often contradictory ways through history. It has been used to describe the symptoms suffered following a head injury as well as the pathophysiological mechanisms causing these symptoms^7^ (see ref 17 for a detailed history of the use of the term concussion). The modern use of the term reflects these factors.

**Figure 1:**
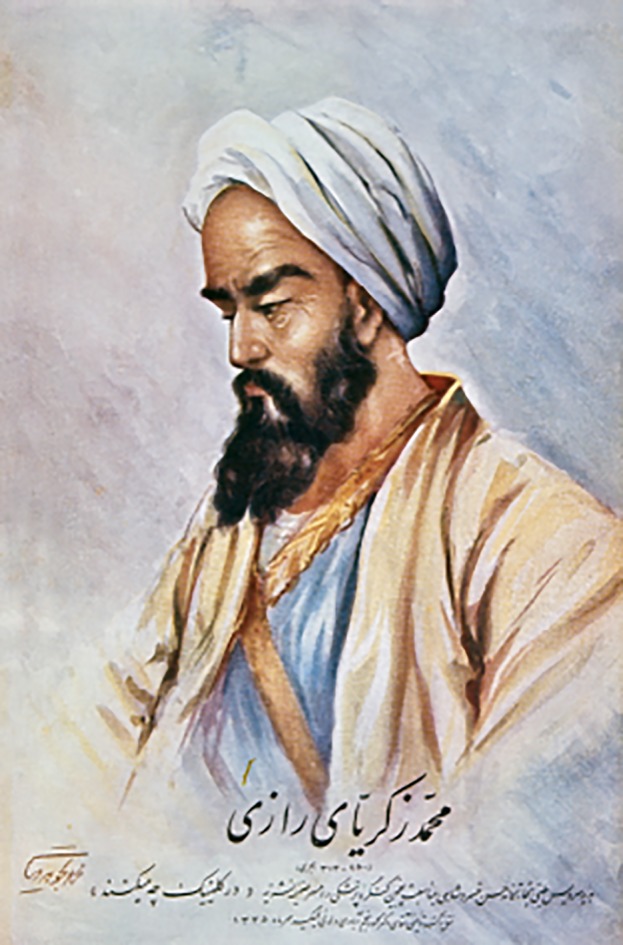
The Persian physician Razes.

The first use of the term in a modern context probably occurred towards the end of the first millennium. The Persian physician Razes (figure 1) used the term concussion to describe an abnormal physiological state of the brain, giving it a specific meaning and separating it from severe brain injury.^17^ In the 13th century Lanfrancus separated commotio cerebri and contusio cerebri, the former referring to a transient disruption of cerebral function brought about by ‘shaking’ of the brain and the latter to overt structural brain damage in the form of contusions or bruising.

This separation continued into the 20th century with the Committee to Study Head Injury Nomenclature proposing the following definition for concussion in 1966:^18^

A clinical syndrome characterised by immediate and transient impairment of neural function, such as alteration of consciousness, disturbance of vision, equilibrium, etc, due to mechanical forces.

However, some authors around this time recognised that not all patients recovered spontaneously and that symptoms could persist. For example, Symonds proposed that concussion should include those patients in whom residual symptoms persisted,^19^ which he attributed to diffuse nerve cell damage produced at the moment of injury.

## Current definitions of concussion

There is still no universal consensus regarding the definition of concussion. The 2012 Zurich Consensus Statement on Concussion in Sport proposed that concussion and mild TBI should be viewed as distinct entities.^20^ The group defined concussion as a “complex pathophysiological process affecting the brain”, and allowed for the presence of neuropathological damage. However, concussive symptoms were largely thought to reflect a functional disturbance, typically resolving spontaneously with no imaging abnormality. In contrast, recent American Academy of Neurology guidelines for sports concussion in 2013 do not separate concussion from mild TBI, defining concussion as “a clinical syndrome of biomechanically induced alteration of brain function, typically affecting memory and orientation, which may involve loss of consciousness”. However, they noted a lack of consensus in the use of the term, with an overlap in the use of concussion and mild TBI.^21^

Therefore, concussion is currently used in two main ways: (1) to describe a distinct pathophysiological entity with its own diagnostic and management implications, mainly seen in the context of sporting injuries; and (2) to describe a constellation of symptoms that arise after different types of TBI.

## The problems with concussion

It is commonly assumed that patients with postconcussive symptoms are unlikely to have significant structural brain injury. However, the true pathological situation is often much more uncertain. There are two main mechanisms of acute injury in TBI: direct contact and acceleration/deceleration. An object striking the head or the brain striking the inside of the skull produces a direct injury. Alternatively, rapid acceleration and deceleration imparts shear, and tensile and compressive strains that mainly damage long-distance white matter connections by producing diffuse axonal and vascular injury. Primary injury produces skull fractures, intracranial haematoma and diffuse injuries. In addition, secondary injury results from processes triggered by the initial injury, such as ischaemia, raised intracranial pressure, infection and inflammation. Primary and secondary injuries interact to produce a complex pattern of evolving damage.

**Figure 2:**
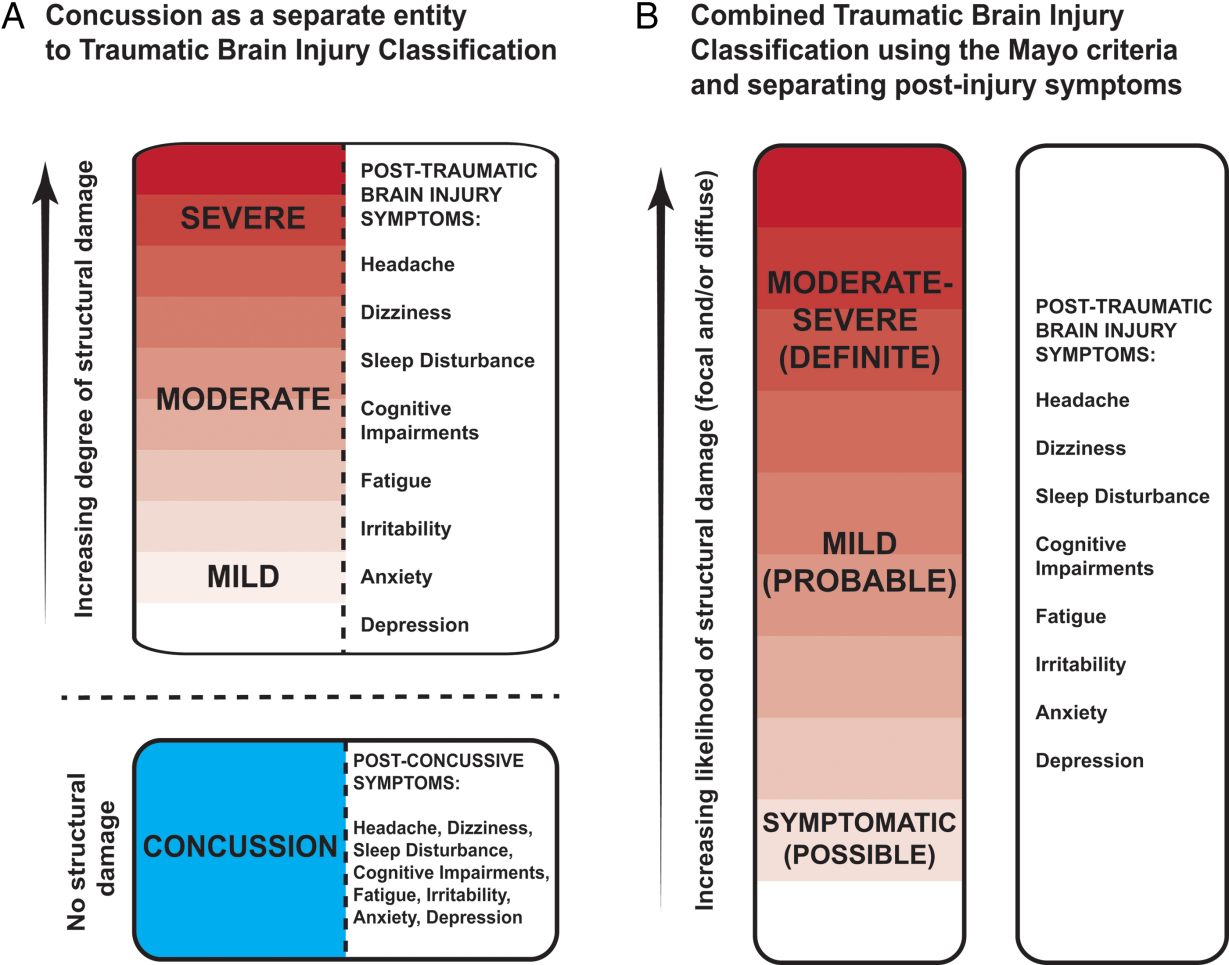
Two potential classification systems for traumatic brain injury and concussion.

In this context, separating concussion as a distinct pathophysiological entity is very problematic (figure 2A). There is no clear pathological definition to distinguish concussion from other types of TBI, and the injuries leading to concussion are biomechanically similar to other types of TBI. Therefore, there is no a priori reason to think that concussion and mild TBI could be distinguished pathologically. It is also unclear how a clinician might decide between mild TBI and concussion, as the symptoms and signs of concussion also follow other types of TBI. For example, headache, cognitive impairment, emotional lability, loss of consciousness and sleep disturbance, each occur to variable extents after all types of TBI. Therefore, it is futile to try to separate concussion as a distinct entity on clinical grounds.

There are also problems in retaining concussion as a diagnostic label for the constellation of symptoms that are commonly experienced after TBI. Here concussion usually implies a ‘benign’ set of problems that will eventually resolve spontaneously. However, the assumed transience of ‘concussion’ symptoms is problematic, as many patients do not recover quickly and it is difficult to predict long-term outcome after TBI. Even apparently trivial injuries can sometimes have long-term effects, with patients reporting similar postconcussive symptoms after TBI of all severities.^8^ This can result in a circularity in diagnosis and prognostication. It is easy for the neurologist, who often has limited access to information about the nature of the original injury, to assume that a constellation of ‘postconcussive’ symptoms is likely to be benign—because of their ‘postconcussive’ nature. This can obviously result in patients being inappropriately reassured that their symptoms will spontaneously resolve, as well as a lack of investigation and treatment.

Standard investigations also do not particularly help in defining ‘concussion’. Many patients with mild TBI do not undergo neuroimaging and are perhaps wrongly reassured about the concussive nature of their problems without any detailed investigation. Even when there is available neuroimaging, it is easy to be falsely reassured by negative neuroimaging findings. Standard neuroimaging will identify large focal contusions or haemorrhage but normal conventional CT and MRI do not exclude diffuse axonal and vascular injury, both major drivers of poor clinical outcome after TBI (see Investigation section). Standard neuroimaging sequences can miss these problems, although more advanced techniques such as susceptibility weighted and diffusion MRI are a more sensitive way of identifying them.^22^ ^23^

Finally, the term concussion lacks any diagnostic precision and at worst encourages a lazy diagnostic approach. Arriving quickly at the diagnosis of ‘postconcussive syndrome’ often curtails a detailed assessment of the post-traumatic symptoms. For example, patients with migrainous headaches may be labelled as having concussion, and denied more accurate diagnosis and treatment. This is a type of diagnostic bias where undue emphasis is placed on one aspect of the presentation (the initial injury), which has the effect of obscuring other elements of the diagnostic process. As a result patients with disabling problems often feel that they have not been properly assessed, ‘not been listened to’ and are ‘not getting the services that their injuries deserve’.^24^ This type of ‘broad-brush’ approach to the neurological assessment of patients with TBI is often justified by therapeutic nihilism. We believe such pessimism is inappropriate and that patients with TBI can benefit from the same level of diagnostic precision and careful management that neurologists bring to other areas of their practice.

## What should replace concussion?

We propose that the terms concussion and postconcussion syndrome are unhelpful and should be ‘retired’. Instead, we should use a unified classification of the severity of TBI, coupled with a careful attempt to identify the underlying cause for any persistent post-traumatic symptoms (figure 2B). There are several TBI severity classification systems, but we recommend using the Mayo system (see box 1).^25^ This uses traditional estimates of severity based on loss of consciousness duration, Glasgow coma scale score and post-traumatic amnesia duration, and incorporates neuroimaging measures of injury severity. It separates the large group of patients with mild TBI into two groups, referred to as mild (probable) and symptomatic (possible) TBI. This distinction is useful as it acknowledges the heterogeneity of mild TBI, and makes it explicit that there is wide variation in the likelihood of significant neuropathology across the subgroup. For simplicity we shall refer to these two groups collectively as ‘mild TBI’ for the remainder of this article.

Box 1 **Mayo Traumatic Brain Injury (TBI) Classification System**

- Classify as *Moderate–Severe (Definite) TBI* if one or more of the following criteria apply:
  1. Death due to this TBI
  2. Loss of consciousness of 30 min or more
  3. Post-traumatic anterograde amnesia of 24 h or more
  4. Worst Glasgow Coma Scale full score in first 24 h <13 (unless invalidated upon review eg, attributable to intoxication, sedation, systemic shock)
  5. One or more of the following present:
    - Intracerebral haematoma
    - Subdural haematoma
    - Epidural haematoma
    - Cerebral contusion
    - Haemorrhagic contusion
    - Penetrating TBI (dura penetrated)
    - Subarachnoid haemorrhage
    - Brainstem injury
- If none of Criteria A apply, classify as *Mild (Probable) TBI* if one or more of the following criteria apply:
  1. Loss of consciousness momentarily to less than 30 min
  2. Post-traumatic anterograde amnesia momentarily to less than 24 h
  3. Depressed, basilar or linear skull fracture (dura intact)
- If none of Criteria A or B apply, classify as *Symptomatic (Possible) TBI* if one or more of the following symptoms are present:
  - Blurred vision
  - Confusion (mental state changes)
  - Daze
  - Dizziness
  - Focal neurological symptoms
  - Headache
  - Nausea

In the future, additional factors may also help prognostication. Large studies of clinical outcome after TBI are underway, such as Centre TBI (https://www.center-tbi.eu/), which will help to define the key factors determining clinical outcome. The risks of developing Alzheimer's disease^26^ and Parkinson's disease^11^ are already known to be related to apolipoprotein EACE-R (APOE) and α-synuclein genotypes, respectively, and in the future genetic factors should allow a personalised calculation of the risks of a poor clinical outcome.

## A hierarchical approach to management of mild TBI

**Figure 3:**
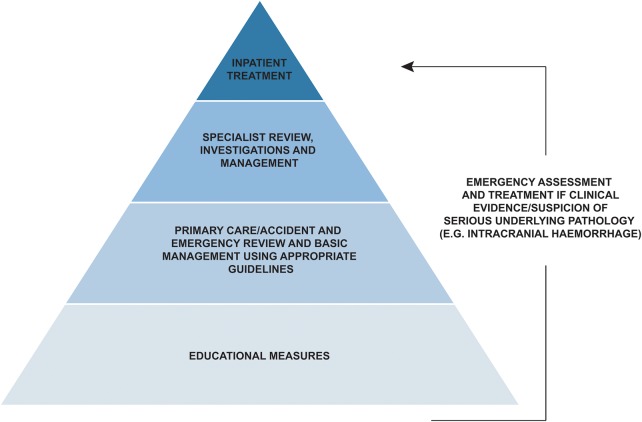
A hierarchical approach to the management of mild traumatic brain injury.

As head injuries are very common and most patients improve spontaneously, we need a hierarchical approach to medical management of mild TBI (figure 3).

### Emergency assessment

Patients sometimes require emergency treatment, even after apparently minor injuries. The UK National Institute of Health and Care Excellence (NICE) guidelines outline clinical red flags,^27^ providing guidance about when this is necessary (see box 2 for NICE guidelines regarding acute CT imaging after TBI). The acute hospital management of TBI has recently been reviewed in *Practical Neurology* and we will not discuss this further here.^28^

Box 2 **National Institute of Health and Care Excellence (NICE) Guidelines for determining the need for an acute CT scan of the head in adults following a traumatic head injury**

CT scan of head within 1 hour if any of the following are present:

- Glasgow Coma Scale (GCS) score <13 on initial assessment
- GCS<15 2 hours after injury
- Suspected open or depressed skull fracture
- Any sign of basal skull fracture
- Post-traumatic seizure
- Focal neurological deficit
- >1 episode of vomiting since the head injury

CT scan of head within 8 hours if:

- Current warfarin treatment
- Loss of consciousness or amnesia and any of the following:
  Age >65 years
  A history of bleeding or clotting disorder
  Dangerous mechanism of injury (a pedestrian or cyclist struck by a motor vehicle, an occupant ejected from a motor vehicle or a fall from height of more than 1 m or five stairs)
  More than 30 min retrograde amnesia of events immediately before the head injury

### Educational measures and community management

There is considerable confusion about how to manage mild TBI in the community, as well as wide variations between countries. This variability is exemplified in the management of sporting head injuries. Mild TBI is common in the context of contact sports, but there is variable advice for players, parents and professional bodies. Following several high profile litigation cases in the USA, particularly in the National Football League, clear guidelines have been drawn up.^29^ Unusually, the USA has primary legislation linked to these guidelines for athletes aged under 18 years, the Zackery Lystedt law. This stipulates a requirement for education around concussion, clear return to play rules, and when it is required for a player to be reviewed by a healthcare professional with expertise in TBI.

**Figure 4:**
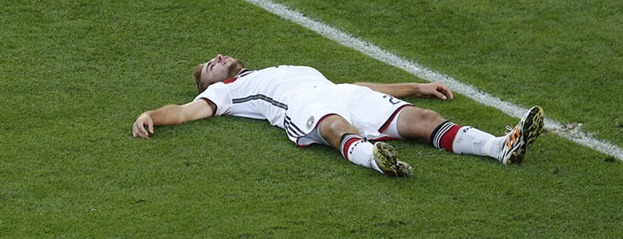
A football player knocked unconscious at the World Cup 2014. He played on for a further 14 minutes before being substituted (see figure 5).

**Figure 5:**
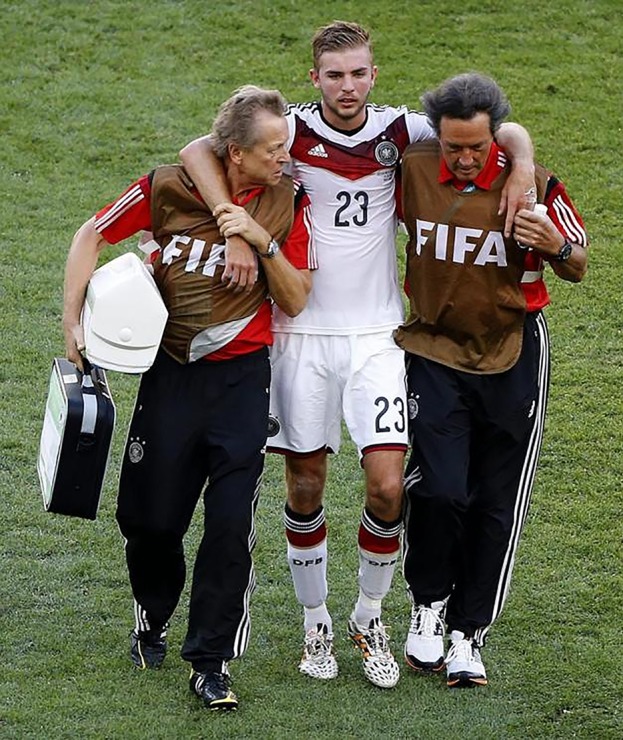
The football player from figure 4 is led off having played on for 14 minutes after being knocked unconscious.

Outside the USA there is often a lack of clear guidelines or a failure to enforce them.^30^ This confusion was illustrated at the 2014 football World Cup where three players lost consciousness after head injury and continued to play despite being clearly unfit (figures 4 and 5).^30^ Some sports have engaged more actively in the need to develop clear guidelines (eg, see the World Rugby guidelines^31^). There is considerable value in developing consensus guidelines that apply across sports, as it is challenging to communicate and implement simple advice at the ‘pitchside’ and this is hampered by inconsistency. In the UK, the Faculty of Sports and Exercise Medicine is currently reviewing this subject.

In general, simple educational measures can reduce symptom duration and severity after mild TBI.^32^ ^33^ Providing an information booklet detailing common symptoms and coping strategies with a single follow-up session helps to reduce persistent symptoms.^34^ There is on-line information available: for example, the Headway charity and Rugby Football Union websites (https://www.headway.org.uk/ and http://www.englandrugby.com/my-rugby/players/player-health/concussion-headcase/). Due to the large numbers of mild TBI, it is too demanding of resources to provide a medical follow-up visit in many situations. Alternative approaches include telephone follow-up^35^ and/or routinely following up only those patients felt most likely to have poor outcome.

### Primary/emergency department care

Initial medical assessment in the UK usually takes place either by general practitioners or in emergency departments. Awareness of the effects of mild TBI varies widely in these contexts. Improved guidance and education particularly aimed at GPs and emergency doctors is likely to help. Major trauma centres within the UK have centralised the management of TBI in the hospital setting. This is driving significant improvements in the emergency care management of TBI, although most focus is on moderate/severe injuries; patients with mild TBI still often receive inconsistent advice.

### Specialist review

Only a small proportion of patients with mild TBI in the UK is reviewed by a neurologist or another TBI specialist. However, patients often benefit from specialist review when symptoms persist. Systematic outpatient follow-up can help the functional outcome after head injury.^32^ This should ideally be provided in the context of a multidisciplinary team because the long-term effects of TBI are often multifactorial. Important inputs can be provided from neuropsychiatrists, psychologists, physiotherapists, endocrinologists, nurse specialists, vestibular specialists and occupational therapists. Some patients require readmission to hospital after their acute management, particularly those with very significant cognitive and psychiatric problems. It can be difficult to know when to escalate assessment and intervention, and funding can be problematic to secure. Nevertheless, the presence of severe cognitive impairment, uncontrolled epilepsy, violent tendencies, family breakdown, alcohol and drug abuse, and homelessness should trigger consideration of specialist inpatient rehabilitation. Patients diagnosed with ‘postconcussion’ syndrome are less likely to be referred for specialist review, but access to detailed assessment should not be determined by an uninformative diagnostic label.

## Investigating mild TBI

### Neuroimaging

Standard structural brain CT and MRI are key investigations for the assessment of TBI (see box 2 for CT guidelines). CT is sensitive to skull fractures, focal brain injury and intracerebral bleeding and allows the identification of patients who have a moderate/severe injury according to the Mayo criteria.^25^ However, standard neuroimaging is often insensitive to subtle vascular or white matter injuries such as diffuse axonal injury, which can be seen in mild TBI. Therefore, a normal CT or standard MRI can be falsely reassuring.

**Figure 6:**
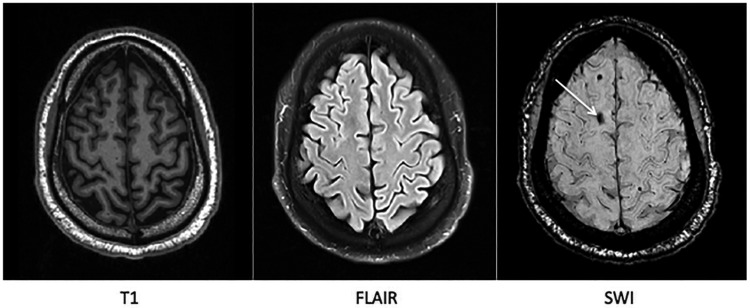
A microbleed is clearly identified on susceptibility weighted MRI (marked with white arrow) but not clearly visible on standard T1 weighted nor fluid-attenuated inversion recovery MRI.

**Figure 7:**
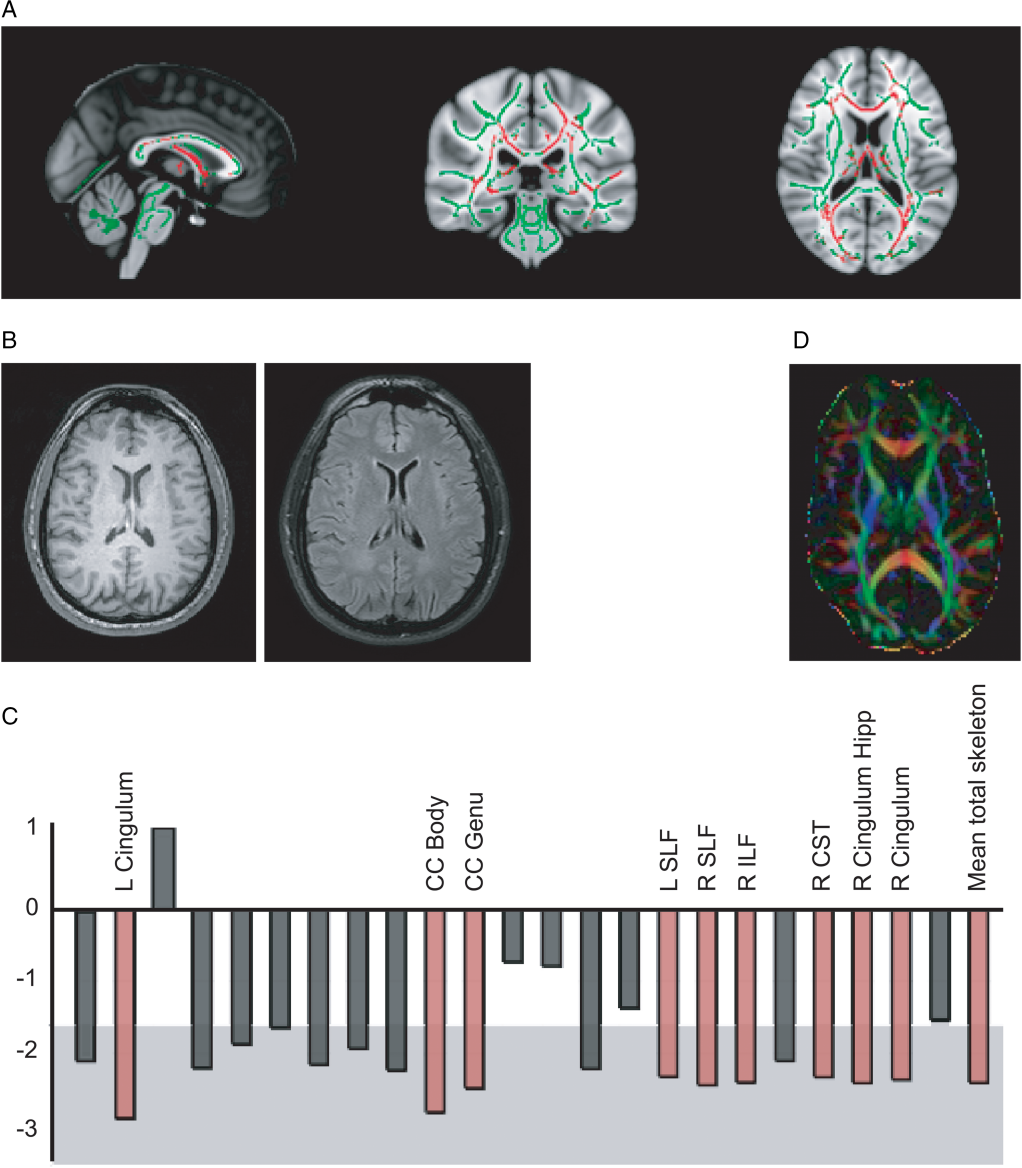
(A) Diffusion-tensor imaging assessment of white matter damage after traumatic brain injury (TBI). Axial images show a contrast between mild TBI and control groups. Normal white matter is shown in green, with red regions showing damaged areas (low fractional anisotropy).^23^ (B) and (C) A single case study of a 41-year-old man with a mild TBI following a road traffic collision (post-traumatic amnesia of <24 h, loss of consciousness <30 min). (B) Normal structural MRI (T1 and fluid-attenuated inversion recovery). (C) Diffusion-tensor imaging assessment of white matter structure. The graph shows Z-scores for the comparison of fractional anisotropy in each tract between the patient and controls. The central white area denotes the area of Z<1.64 (p>0.01) for the control group’s fractional anisotropy. Red bars indicate where that tract's fractional anisotropy value was >2.3 SDs from the control group mean. This provides evidence for extensive damage throughout this patient's white matter, despite normal standard structural imaging. (D) An illustration of diffusion-tensor imaging data, where the colour represents the predominant direction of water diffusion. L, left; R, right; CC, corpus callosum; SLF, superior longitudinal fasciculus; ILF, inferior longitudinal fasciculus; CST, corticospinal tract; Hipp, hippocampus.

Diffuse axonal and vascular injuries are important factors in producing poor clinical outcome.^36–39^ More advanced MRI techniques are sensitive to these effects. Gradient-echo and susceptibility weighted imaging can show microbleeds (figure 6), which are a stable marker of white matter injury after TBI.^40^ Susceptibility weighted imaging should now form part of the routine radiological investigation of TBI. In addition, diffusion MRI can provide a more complete and quantified assessment of white matter structure. This has been widely used in a research setting but is not yet widely available clinically.^41^ Diffusion-tensor imaging quantifies the diffusion characteristics of water (figure 7). These are altered by changes in tissue microstructure, providing a sensitive marker of white matter injury.^23^ ^42^ ^43^ Diffusion MRI can help predict clinical outcome^44^ and in mild TBI the extent of diffusion changes correlates with cognitive impairment.^23^ ^45–47^

Nuclear medicine imaging and functional MRI can provide evidence of physiological changes following mild TBI. Single-photon emission CT can show alterations in cerebral blood flow after mild TBI despite normal CT scans.^48–50^ Functional MRI also measures changes in blood flow and can show abnormalities following mild TBI that relate to cognitive function.^51^ ^52^ These types of imaging are largely confined to a research setting but hold promise for future clinical use.

Positron emission tomography allows markers of neurodegeneration such as β-amyloid and tau pathology as well as markers of inflammation to be seen in vivo. Positron emission tomography ligands sensitive to β-amyloid show increased distribution volumes in patients who have suffered a TBI^53^ and ligands sensitive to activated microglia suggest persistent inflammatory responses up to 6 years following a TBI.^54^ These techniques have yet to be used in patients who have suffered mild or repetitive head injuries but offer promise in the future for detecting evidence of neurodegeneration and the potential mechanisms driving it, such as inflammation.

### Blood and cerebrospinal fluid investigations

Hypothalamo-pituitary dysfunction should be screened for in patients with persistent symptoms.^55^ Endocrine abnormalities are common in the acute phase, but often resolve quickly. However, 10–50% of patients with TBI may be persistently affected, with growth hormone deficiency most common.^56–58^ Our own experience suggests that in civilian TBI the true rate of endocrine problems is closer to 10% and is likely to be lower following mild TBI. Nevertheless, appropriate hormonal replacement can improve post-TBI symptoms. One screening approach is to take baseline pituitary blood tests in all patients with persistent symptoms. Testing around 3 months after injury allows time for acute dysfunction to resolve. This approach provides information about hypothalamo-pituitary function, although expert endocrine input and dynamic testing will be necessary to completely exclude impairments.

Recent research has attempted to find blood and cerebrospinal fluid (CSF) biomarkers of TBI.^59^ Markers of neuronal damage such as total tau and neurofilament light polypeptide are elevated acutely in the CSF, with levels correlating with the exposure to head injury.^60^ ^61^ Measuring CSF is, however, unlikely to be practical following mild TBI and therefore we need a blood biomarker. Unfortunately, the evidence for a blood biomarker is less convincing than for CSF,^59^ although a recent study showed correlation between the total tau levels in plasma acutely following a mild head injury and the time taken for symptoms to resolve in ice-hockey players.^62^

## Managing post-traumatic symptoms

### Cognitive impairment

Cognitive impairment resolves rapidly in most patients with mild TBI. However, significant numbers of patients present to neurologists with disabling cognitive symptoms after mild TBI, and the underlying cause can be challenging to evaluate. In general, the most common impairments are in the domains of awareness, processing speed, memory, attention and executive function,^63–67^ although their prevalence after mild TBI is debated. Persistent impairments of information processing speed, attention, memory and executive domains occur in some^68–71^ but not all studies^72–77^ reflecting the heterogeneity of mild TBI. In this context, cognitive testing is key to identifying objective deficits. Formal neuropsychometric assessment administered by a clinical psychologist is ideal. Assessments of effort can be particularly useful, as this may be reduced in situations where there is secondary gain such as medical litigation. However, detailed neuropsychometric assessment is not always practical even for patients being evaluated in a specialist setting, but all patients should have some form of cognitive testing. Traditional cognitive screening tests such as the Mini-Mental State Examination or the longer Addenbrooke's Cognitive Examination^78^ can provide useful information in the clinic, although they are not optimal for evaluating cognition after TBI. These traditional ‘paper and pencil’ screening test are most useful for memory assessment after TBI but are less effective at identifying impairments of attention, processing speed and executive function.

Computerised assessment of cognition is increasingly used, especially for sports injury.^79^ For example, professional rugby players typically complete preseason cognitive screening and are reassessed following TBI and just before their anticipated return to play. This can be a powerful approach, although care needs to be taken in interpreting results as players sometimes attempt to ‘game’ the system by deliberately performing poorly in baseline assessments.^80–82^ More generally, longitudinal assessment should allow cognitive function to be sensitively tracked after TBI and its routine use should now be feasible at low cost. Our current approach in clinic is to screen all new patients with the Addenbrooke's cognitive examination-revised (ACE-R), combined with computerised assessment of the domains commonly affected by TBI. We are evaluating internet-based longitudinal tracking of cognition and complex patients are then referred on for formal neuropsychometry.

Catecholaminergic and cholinergic agents can enhance cognition after TBI^83^ ( for review). The best evidence is for methylphenidate and amantadine. At least 15 trials (10 were randomised-controlled trials, RCTs) have investigated methylphenidate as a cognitive enhancer, although all are relatively small (N=40 or fewer). Most indicate that it leads to faster information processing.^84–86^ Less consistently, there were improvements in functional outcomes and attentional measures.^84^ ^86^ ^87^ Meta-analysis evidence also suggests that methylphenidate can improve anger, aggression and psychosocial function.^88^ Most of these studies focus on moderate to severe injuries but two trials reported a benefit in mild to moderate traumatic brain injuries.^89^ ^90^ Amantadine is an indirect dopamine agonist and N-methyl-D-aspartate (NMDA) antagonist, and two double-blind RCTs support its use in the first 6 months after severe TBI.^91^ ^92^ A recent large multicentre international RCT (N=184) showed it accelerated recovery over the first 4 months after TBI,^91^ with improvements across all behavioural measures including sustained attention, command following and object recognition. In addition, there is also evidence that the cholinergic agent donepezil, widely used to treat memory disturbance in Alzheimer's disease, can enhance memory and attention following TBI.^88^

Approaches to cognitive rehabilitation include extensive practice, and training patients to try and compensate for impairments by using preserved cognitive abilities. These techniques typically improve performance on measures similar to the tasks trained on, with as yet a lack of persuasive evidence that this translates to marked improvements in day-to-day functioning.^93^ Cognitive rehabilitation works best when incorporated into a well-supported rehabilitation programme,^94^ ^95^ although outside a few specific contexts (eg, the Headley Court mild TBI military rehabilitation programme) this does not exist in the UK for mild TBI (table 1).

**Table 1:** Managing post-traumatic symptoms

Symptom	Diagnosis	Treatments
Cognitive impairments	General Measures	Cognitive rehabilitation,^96^ ideally in the context of a holistic rehabilitation programme. Computer based neuropsychology training^97–99^ Treat underlying depression^100^ ^101^ Treat underlying sleep disturbance Treat underlying endocrine disturbance
	Pharmacological treatments	Dopaminergic medications: for example, methylphenidate and amantadine^83^ Cholinergic medications: for example, donepezil and rivastigmine^83^
Psychiatric problems	Depression Anxiety	Psychological therapies for example, cognitive-behaviour therapy^102^ ^103^ Medications: SSRIs (in particular sertraline and citalopram)^104–106^ Education Treat underlying sleep disturbance Treat underlying endocrine disturbance
Headache	Migraine or probable migraine	Treatment as for primary migraine (including lifestyle measures, acute and prophylactic treatment)^107^ (consider greater occipital nerve injection^108^)
	Tension type headache	Simple analgesics. Prophylactic treatment, for example, amitriptyline or alternative tricyclic antidepressant
	Cervicogenic	Physiotherapy^109^ Nerve blocks^110^
	Medication overuse	Reduce medication overuse. Avoid long-term use of opiates. Simple analgesics no more than two headache days per week.
Dizziness	Benign paroxysmal positional vertigo	Repositioning manoeuvres.^111^ Physiotherapy
	Migrainous vertigo	Migraine treatment as above, first-line propranolol.
	Central vestibular system problems (ie, injury to the central nervous system sections of the vestibular system)	Vestibular rehabilitation^112^
	Non-specific post-traumatic dizziness	Vestibular rehabilitation^112^
Sleep disturbance	Insomnia	Cognitive behavioural therapy for insomnia^113^ Sleep hygiene measures (eg, remove electronic equipment from bedroom, reduce light and noise disturbance, etc) Nocturnal hypnotics (use with caution due to risk of impairing cognitive functions)^114^
	Obstructive sleep apnoea	Continuous positive airway pressure
	Daytime sleepiness	Modafinil^115^
Fatigue		Treat underlying depression Treat underlying sleep disturbance Treat underlying endocrine disturbance^116^ Bright light therapy^117^ Physical conditioning programmes^118^ ^119^

SSRI, selective serotonin reuptake inhibitor.

### Psychiatric symptoms

Although psychiatric symptoms are common after TBI, patients can find it difficult to access specialists who view them as ‘their problem’. They often coexist with cognitive impairment and can influence clinical outcome, interfere with rehabilitation and increase mortality.^120–123^ New psychiatric problems often occur after injury,^124^ either through direct neural effects or because of a psychological reaction to the impact of the injury. However, pre-existing mental health disorders increase the risk of developing a psychiatric disorder after injury (∼75% vs 45%).^124^ Depression is particularly common^122^ ^125^ and can be treated with selective serotonin reuptake inhibitors and tricyclic antidepressants^83^ (for review). Selective serotonin reuptake inhibitors are favoured because of the potential for tricyclic antidepressants to impair cognitive function.^126^ Sertraline and citalopram have the greatest evidence base^104–106^ and we favour their use in our clinic. Psychological treatments may also be helpful, with a recent RCT finding mindfulness training reduced symptoms of post-TBI depression.^102^ For anxiety, including post-traumatic stress disorder, psychological treatment with cognitive behavioural therapy is likely to help.^103^ One RCT found it helped to prevent post-traumatic stress disorder after mild TBI^127^ and another reported that a combination of cognitive behavioural therapy and neurorehabilitation reduced anxiety symptoms after TBI.^128^ Our practice is to view psychiatric problems as being within the neurological remit. We therefore routinely assess and treat psychiatric problems as part of our neurological practice, working closely with a neuropsychiatrist who advises about optimal treatment approaches in a multidisciplinary team setting.

### Headache

Head injuries often produce headache.^129^ ^130^ The duration is very variable and often does not relate clearly to injury severity.^129^ ^131^ The pathogenesis of post-traumatic headache is poorly understood. Local trauma or muscular injuries presumably account for some headaches but many cases of mild head injury do not produce obvious focal injury. Experimental mild TBI does, however, show similar biochemical changes as that seen in migraine, suggesting a possible pathogenic mechanism to explain the high incidence of post-traumatic headache.^132^ Analgesic treatment is effective acutely, using combinations of opiates, paracetamol and non-steroidal anti-inflammatory drugs. The need for these medications usually rapidly reduces over the first few weeks. Once the initial period of acute pain has settled (usually within the first 2 months), we advise patients to avoid opiate medication completely and to taper other analgesics to avoid analgesic overuse headache.

It is surprisingly common for headaches to persist for many months after mild TBI.^130^ A recent prospective study of more than 200 patients found a 1-year cumulative incidence of 91%, with migraine present in 50%.^130^ A reactivation or worsening of migraine frequency is common, and migrainous type headaches sometimes occur de novo. Previous history of migraine, age <60 years, female sex, and mood disturbance have been associated with more persistent headaches.^129^ ^131^ Tension-type headache and cervicogenic headache are also common.^130^ ^133^

Given the incidence of headache and the risk of chronic pain, a diagnosis of ‘post-concussion’ headache is unhelpful, as it is often accompanied by a failure to manage the problem actively. It is unclear whether treatment guidelines can be extrapolated from primary headache disorders. However, in our experience symptomatic migraine treatment with non-steroidal anti-inflammatory drugs and triptans can be helpful, and prophylactic medication is often required for frequent headaches with propranolol and amitriptyline both sometimes effective. There is limited clinical trial evidence available, although triptans and topiramate are effective.^134^ It is useful to consider the presence of other symptoms when choosing prophylactic treatment. For example, if patients are anxious or require a mood stabiliser, propranolol or sodium valproate may treat the headache and mood problems. In contrast, if there is an element of insomnia then amitriptyline is a sensible choice. Physiotherapy or nerve blocks can help for headaches of cervicogenic origin.^109^ ^110^ From our experience and in a small number of reported cases, greater occipital nerve injections can help to treat post-traumatic headaches, especially if there is localised tenderness.^108^

### Dizziness

Dizziness affects up to 80% of patients in the first few days following a head injury.^135^ It often persists after mild head injury, with up to a fifth of patients still symptomatic 5 years later.^136^ This is often labelled as ‘post-concussion’ dizziness, but this is not a useful ‘diagnosis’. In acute head injury, expert neuro-otological review can identify the causes for dizziness in virtually all cases (personal communication B Seemungal), with the most common diagnoses being benign paroxysmal positional vertigo, migraine and damage to the central vestibular system, which can produce vestibular agnosia.^137^ There are distinct treatments for most common vestibular conditions, and a pragmatic approach to management was recently described in *Practical Neurology*,^138^ an approach relevant to patients with TBI.

Benign paroxysmal positional vertigo is the most common cause of dizziness after TBI, presumably because of the mechanical effect of the injury on semicircular canal function. It can be diagnosed with Hallpike's testing and treated via repositioning manoeuvres.^139^ However, post-traumatic benign paroxysmal positional vertigo can be challenging for the non-expert as it can involve multiple canals and may even be bilateral, explaining why a single treatment session may not suffice.^140^ Following effective therapy the long-term relapse rate is no different from that in idiopathic benign paroxysmal positional vertigo (∼15%).^111^ Intermittent episodes of vertigo associated with headaches or other migrainous features should alert the neurologist to the possibility of migrainous vertigo. Our experience is that this can be successfully treated using generic approaches to migraine treatment with propranolol being our first choice. Vestibular rehabilitation helps in unselected cases of vertigo and balance impairment after mild TBI.^112^ Anxiety is common in chronic dizziness and the combination is associated with worse outcome.^141^ Hence, combining cognitive behavioural therapy for anxiety with vestibular rehabilitation is likely to be effective.

### Sleep disturbance

Sleep disturbance is also very common after TBI. Questionnaires can be used to screen for significant problems,^142^ ^143^ although polysomnography will often help clarify the cause of disturbance. Several factors can impair sleep following TBI including chronic pain, depression, obstructive sleep apnoea^144^ and impaired endogenous melatonin production.^145^ Simple measures such as encouraging basic sleep hygiene can be helpful. Obstructive sleep apnoea can be managed with non-invasive ventilation techniques and there may be a role for melatonin treatment. Modafinil is useful for treating excessive daytime sleepiness, although it may not lead to improvements in the associated cognitive problems.^115^ Benzodiazepines and gamma-Aminobutyric acid (GABA) agonists can exacerbate cognitive impairments, so should be avoided or used with caution.^114^ Cognitive behavioural therapy for insomnia is likely to be an effective non-pharmacological approach to insomnia, and a small study showed improved sleep and reduced fatigue after a brief intervention in patients with TBI.^113^

## Conclusions

We propose that the term concussion should be retired because it has no clear and consistently understood definition, leads to diagnostic confusion and can limit the use of effective treatments of post-traumatic problems. Instead, neurologists should adopt a single classification system for all TBI based on injury severity and attempt a precise diagnosis of post-traumatic problems. It is important to recognise that mild TBI is not always a benign condition, and patients sometimes fail to recover from what may appear to be innocuous injuries. It is difficult to predict clinical outcome and interventions can reduce the burden of disability after TBI. Therefore, neurologists should review more patients with TBI and intervene more actively.

## References

[1] Kay A, Teasdale G Head injury in the United Kingdom. World J Surg 2001;25:1210–20. 10.1007/s00268-001-0084-6 11571960

[2] Alexander MP Mild traumatic brain injury: pathophysiology, natural history, and clinical management. Neurology 1995;45:1253–60. 10.1212/WNL.45.7.1253 7617178

[3] Kashluba S, Paniak C, Blake T, et al A longitudinal, controlled study of patient complaints following treated mild traumatic brain injury. Arch Clin Neuropsychol 2004;19:805–16. 10.1016/j.acn.2003.09.005 15288333

[4] Ponsford J, Willmott C, Rothwell A, et al Factors influencing outcome following mild traumatic brain injury in adults. J Int Neuropsychol Soc 2000;6:568–79. 10.1017/S1355617700655066 10932476

[5] Hou R, Moss-Morris R, Peveler R, et al When a minor head injury results in enduring symptoms: a prospective investigation of risk factors for postconcussional syndrome after mild traumatic brain injury. J Neurol Neurosurg Psychiatry 2012;83:217–23. 10.1136/jnnp-2011-300767 22028384

[6] Norrie J, Heitger M, Leathem J, et al Mild traumatic brain injury and fatigue: a prospective longitudinal study. Brain Inj 2010;24:1528–38. 10.3109/02699052.2010.531687 21058899

[7] Stulemeijer M, van der Werf S, Borm GF, et al Early prediction of favourable recovery 6 months after mild traumatic brain injury. J Neurol Neurosurg Psychiatry 2008;79:936–42. 10.1136/jnnp.2007.131250 17951281

[8] Ryan LM, Warden DL Post concussion syndrome. Int Rev Psychiatry 2003;15:310–16. 10.1080/09540260310001606692 15276952

[9] Gouvier WD, Cubic B, Jones G, et al Postconcussion symptoms and daily stress in normal and head-injured college populations. Arch Clin Neuropsychol 1992;7:193–211. 10.1016/0887-6177(92)90162-G 14591254

[10] Miller H Accident neurosis. Br Med J 1961;1:992–8. 10.1136/bmj.1.5231.992 13770778 PMC1953243

[11] Goldman SM, Kamel F, Ross GW, et al Head injury, alpha-synuclein Rep1, and Parkinson's disease. Ann Neurol 2012;71:40–8. 10.1002/ana.22499 22275250 PMC3270814

[12] Mayeux R, Ottman R, Maestre G, et al Synergistic effects of traumatic head injury and apolipoprotein-epsilon 4 in patients with Alzheimer's disease. Neurology 1995;45 (3 Pt 1):555–7. 10.1212/WNL.45.3.555 7898715

[13] McKee AC, Stern RA, Nowinski CJ, et al The spectrum of disease in chronic traumatic encephalopathy. Brain 2013;136 (Pt 1):43–64. 10.1093/brain/aws307 23208308 PMC3624697

[14] Baugh CM, Stamm JM, Riley DO, et al Chronic traumatic encephalopathy: neurodegeneration following repetitive concussive and subconcussive brain trauma. Brain Imaging Behav 2012;6:244–54. 10.1007/s11682-012-9164-5 22552850

[15] McMillan TM, Teasdale GM, Weir CJ, et al Death after head injury: the 13 year outcome of a case control study. J Neurol Neurosurg Psychiatry 2011;82:931–5. 10.1136/jnnp.2010.222232 21282727

[16] McMillan TM, Weir CJ, Wainman-Lefley J Mortality and morbidity 15 years after hospital admission with mild head injury: a prospective case-controlled population study. J Neurol Neurosurg Psychiatry 2014;85:1214–20. 10.1136/jnnp-2013-307279 24623794

[17] McCrory PR, Berkovic SF Concussion: the history of clinical and pathophysiological concepts and misconceptions. Neurology 2001;57:2283–9. 10.1212/WNL.57.12.2283 11756611

[18] Congress of Neurological Surgeons. Proceedings of the Congress of Neurological Surgeons in 1964: Report of the Ad Hoc Committee to Study Head Injury Nomenclature. Clin Neurosurg 1966;12:386–94.

[19] Symonds C Concussion and its sequelæ. Lancet 1962;279:1–5. 10.1016/S0140-6736(62)92635-1

[20] McCrory P, Meeuwisse WH, Aubry M, et al Consensus statement on concussion in sport: the 4th International Conference on Concussion in Sport held in Zurich, November 2012. Br J Sports Med 2013;47:250–8. 10.1136/bjsports-2013-092313 23479479

[21] Giza CC, Kutcher JS, Ashwal S, et al Summary of evidence-based guideline update: Evaluation and management of concussion in sports: Report of the Guideline Development Subcommittee of the American Academy of Neurology. Neurology 2013;80:2250–7. 10.1212/WNL.0b013e31828d57dd 23508730 PMC3721093

[22] Shenton ME, Hamoda HM, Schneiderman JS, et al A review of magnetic resonance imaging and diffusion tensor imaging findings in mild traumatic brain injury. Brain Imaging Behav 2012;6:137–92. 10.1007/s11682-012-9156-5 22438191 PMC3803157

[23] Kinnunen KM, Greenwood R, Powell JH, et al White matter damage and cognitive impairment after traumatic brain injury. Brain 2011;134 (Pt 2):449–63. 10.1093/brain/awq347 21193486 PMC3030764

[24] Miller C MTBI Rehabilitation: The Patient's Perspective. Secondary MTBI Rehabilitation: The Patient's Perspective, 1998 http://www.headinjury.com/linktbinih.htm

[25] Malec JF, Brown AW, Leibson CL, et al The mayo classification system for traumatic brain injury severity. J Neurotrauma 2007;24:1417–24. 10.1089/neu.2006.0245 17892404

[26] Mauri M, Sinforiani E, Bono G, et al Interaction between Apolipoprotein epsilon 4 and traumatic brain injury in patients with Alzheimer's disease and Mild Cognitive Impairment. Funct Neurol 2006;21:223–8. 17367583

[27] NICE. Head injury: triage, assessment, investigation and early management of head injury in children, young people and adults. London: National Institute for Health and Care Excellence, 2014. 25340248

[28] Kolias AG, Guilfoyle MR, Helmy A, et al Traumatic brain injury in adults. Pract Neurol 2013;13:228–35. 10.1136/practneurol-2012-000268 23487823

[29] League NF Concussion Protocol. Secondary Concussion Protocol, 2014 http://www.nflevolution.com/concussion-protocol

[30] [No authors listed] Tackling the sports-related concussion crisis. Lancet Neurol 2014;13:747 10.1016/S1474-4422(14)70161-9 25030505

[31] Board IR IRB Concussion Management. Secondary IRB Concussion Management, 2015 http://irbplayerwelfare.com/concussion

[32] Wade DT, Crawford S, Wenden FJ, et al Does routine follow up after head injury help? A randomised controlled trial. J Neurol Neurosurg Psychiatry 1997;62:478–84. 10.1136/jnnp.62.5.478 9153604 PMC486856

[33] Wade DT, King NS, Wenden FJ, et al Routine follow up after head injury: a second randomised controlled trial. J Neurol Neurosurg Psychiatry 1998;65:177–83. 10.1136/jnnp.65.2.177 9703167 PMC2170203

[34] Ponsford J, Willmott C, Rothwell A, et al Impact of early intervention on outcome following mild head injury in adults. J Neurol Neurosurg Psychiatry 2002;73:330–2. 10.1136/jnnp.73.3.330 12185174 PMC1738009

[35] Bell KR, Hoffman JM, Temkin NR, et al The effect of telephone counselling on reducing post-traumatic symptoms after mild traumatic brain injury: a randomised trial. J Neurol Neurosurg Psychiatry 2008;79:1275–81. 10.1136/jnnp.2007.141762 18469027

[36] Hofman PA, Stapert SZ, van Kroonenburgh MJ, et al MR imaging, single-photon emission CT, and neurocognitive performance after mild traumatic brain injury. AJNR Am J Neuroradiol 2001;22:441–9. 11237964 PMC7976823

[37] Hughes DG, Jackson A, Mason DL, et al Abnormalities on magnetic resonance imaging seen acutely following mild traumatic brain injury: correlation with neuropsychological tests and delayed recovery. Neuroradiology 2004;46: 550–8. 15185054 10.1007/s00234-004-1227-x

[38] Lannsjo M, Backheden M, Johansson U, et al Does head CT scan pathology predict outcome after mild traumatic brain injury? Eur J Neurol 2013;20:124–9. 10.1111/j.1468-1331.2012.03813.x 22812542

[39] Smith DH, Meaney DF, Shull WH Diffuse axonal injury in head trauma. J Head Trauma Rehabil 2003;18:307–16. 10.1097/00001199-200307000-00003 16222127

[40] Scheid R, Preul C, Gruber O, et al Diffuse axonal injury associated with chronic traumatic brain injury: evidence from T2*-weighted gradient-echo imaging at 3T. AJNR Am J Neuroradiol 2003;24:1049–56. 12812926 PMC8149043

[41] Ham TE, Sharp DJ How can investigation of network function inform rehabilitation after traumatic brain injury? Curr Opin Neurol 2012;25:662–9. 10.1097/WCO.0b013e328359488f 23108248

[42] Arfanakis K, Haughton VM, Carew JD, et al Diffusion tensor MR imaging in diffuse axonal injury. AJNR Am J Neuroradiol 2002;23:794–802. 12006280 PMC7974716

[43] Rugg-Gunn FJ, Symms MR, Barker GJ, et al Diffusion imaging shows abnormalities after blunt head trauma when conventional magnetic resonance imaging is normal. J Neurol Neurosurg Psychiatry 2001;70:530–3. 10.1136/jnnp.70.4.530 11254782 PMC1737292

[44] Sidaros A, Engberg AW, Sidaros K, et al Diffusion tensor imaging during recovery from severe traumatic brain injury and relation to clinical outcome: a longitudinal study. Brain 2008;131 (Pt 2):559–72. 10.1093/brain/awm294 18083753

[45] Lipton ML, Gellella E, Lo C, et al Multifocal white matter ultrastructural abnormalities in mild traumatic brain injury with cognitive disability: a voxel-wise analysis of diffusion tensor imaging. J Neurotrauma 2008;25:1335–42. 10.1089/neu.2008.0547 19061376

[46] Niogi SN, Mukherjee P, Ghajar J, et al Extent of microstructural white matter injury in postconcussive syndrome correlates with impaired cognitive reaction time: a 3T diffusion tensor imaging study of mild traumatic brain injury. AJNR Am J Neuroradiol 2008;29:967–73. 10.3174/ajnr.A0970 18272556 PMC8128563

[47] Wilde EA, McCauley SR, Hunter JV, et al Diffusion tensor imaging of acute mild traumatic brain injury in adolescents. Neurology 2008;70:948–55. 10.1212/01.wnl.0000305961.68029.54 18347317

[48] Abu-Judeh HH, Parker R, Aleksic S, et al SPECT brain perfusion findings in mild or moderate traumatic brain injury. Nucl Med Rev Cent East Eur 2000;3:5–11. 14600973

[49] Abu-Judeh HH, Parker R, Singh M, et al SPET brain perfusion imaging in mild traumatic brain injury without loss of consciousness and normal computed tomography. Nucl Med Commun 1999;20:505–10. 10.1097/00006231-199906000-00003 10451861

[50] Audenaert K, Jansen HM, Otte A, et al Imaging of mild traumatic brain injury using 57Co and 99mTc HMPAO SPECT as compared to other diagnostic procedures. Med Sci Monit 2003;9:MT112–17. 14523337

[51] Jantzen KJ, Anderson B, Steinberg FL, et al A prospective functional MR imaging study of mild traumatic brain injury in college football players. AJNR Am J Neuroradiol 2004;25: 738–45. 15140712 PMC7974462

[52] Chen JK, Johnston KM, Frey S, et al Functional abnormalities in symptomatic concussed athletes: an fMRI study. NeuroImage 2004;22:68–82. 10.1016/j.neuroimage.2003.12.032 15109998

[53] Hong YT, Veenith T, Dewar D, et al Amyloid imaging with carbon 11-labeled Pittsburgh compound B for traumatic brain injury. JAMA Neurol 2014;71:23–31. 10.1001/jamaneurol.2013.4847 24217171 PMC4084932

[54] Ramlackhansingh AF, Brooks DJ, Greenwood RJ, et al Inflammation after trauma: microglial activation and traumatic brain injury. Ann Neurol 2011;70:374–83. 10.1002/ana.22455 21710619

[55] Tanriverdi F, Unluhizarci K, Kelestimur F Pituitary function in subjects with mild traumatic brain injury: a review of literature and proposal of a screening strategy. Pituitary 2010;13:146–53. 10.1007/s11102-009-0215-x 20037793

[56] Behan LA, Phillips J, Thompson CJ, et al Neuroendocrine disorders after traumatic brain injury. J Neurol Neurosurg Psychiatry 2008;79:753–9. 10.1136/jnnp.2007.132837 18559460

[57] Schneider HJ, Kreitschmann-Andermahr I, Ghigo E, et al Hypothalamopituitary dysfunction following traumatic brain injury and aneurysmal subarachnoid hemorrhage: a systematic review. JAMA 2007;298:1429–38. 10.1001/jama.298.12.1429 17895459

[58] Bondanelli M, De Marinis L, Ambrosio MR, et al Occurrence of pituitary dysfunction following traumatic brain injury. J Neurotrauma 2004;21:685–96. 10.1089/0897715041269713 15253797

[59] Zetterberg H, Smith DH, Blennow K Biomarkers of mild traumatic brain injury in cerebrospinal fluid and blood. Nat Rev Neurol 2013;9:201–10. 10.1038/nrneurol.2013.9 23399646 PMC4513656

[60] Zetterberg H, Hietala MA, Jonsson M, et al Neurochemical aftermath of amateur boxing. Arch Neurol 2006;63:1277–80. 10.1001/archneur.63.9.1277 16966505

[61] Neselius S, Brisby H, Theodorsson A, et al CSF-biomarkers in Olympic boxing: diagnosis and effects of repetitive head trauma. PloS One 2012;7:e33606 10.1371/journal.pone.0033606 22496755 PMC3319096

[62] Shahim P, Tegner Y, Wilson DH, et al Blood biomarkers for brain injury in concussed professional ice hockey players. JAMA Neurol 2014;71:684–92. 10.1001/jamaneurol.2014.367 24627036

[63] Mathias JL, Wheaton P Changes in attention and information-processing speed following severe traumatic brain injury: a meta-analytic review. Neuropsychology 2007;21:212–23. 10.1037/0894-4105.21.2.212 17402821

[64] Kinsella G, Murtagh D, Landry A, et al Everyday memory following traumatic brain injury. Brain Inj 1996;10:499–507. 10.1080/026990596124214 8806010

[65] Scheid R, Walther K, Guthke T, et al Cognitive sequelae of diffuse axonal injury. Arch Neurol 2006;63:418–24. 10.1001/archneur.63.3.418 16533969

[66] Draper K, Ponsford J Cognitive functioning ten years following traumatic brain injury and rehabilitation. Neuropsychology 2008;22:618–25. 10.1037/0894-4105.22.5.618 18763881

[67] Levin H, Kraus MF The frontal lobes and traumatic brain injury. J Neuropsychiatry Clin Neurosci 1994;6:443–54. 10.1176/jnp.6.4.443 7841815

[68] De Beaumont L, Theoret H, Mongeon D, et al Brain function decline in healthy retired athletes who sustained their last sports concussion in early adulthood. Brain 2009;132 (Pt 3):695–708. 10.1093/brain/awn347 19176544

[69] Di Russo F, Spinelli D Sport is not always healthy: executive brain dysfunction in professional boxers. Psychophysiology 2010;47:425–34. 10.1111/j.1469-8986.2009.00950.x 20030763

[70] Ellemberg D, Leclerc S, Couture S, et al Prolonged neuropsychological impairments following a first concussion in female university soccer athletes. Clin J Sport Med 2007; 17:369–74. 10.1097/JSM.0b013e31814c3e3e 17873549

[71] Matser EJ, Kessels AG, Lezak MD, et al Neuropsychological impairment in amateur soccer players. JAMA 1999;282:971–3. 10.1001/jama.282.10.971 10485683

[72] Belanger HG, Spiegel E, Vanderploeg RD Neuropsychological performance following a history of multiple self-reported concussions: a meta-analysis. J Int Neuropsychol Soc 2010;16:262–7. 10.1017/S1355617709991287 20003581

[73] Broglio SP, Ferrara MS, Piland SG, et al Concussion history is not a predictor of computerised neurocognitive performance. Br J Sports Med 2006;40:802–5; discussion 02–5 10.1136/bjsm.2006.028019 16929049 PMC2564398

[74] Echemendia RJ, Putukian M, Mackin RS, et al Neuropsychological test performance prior to and following sports-related mild traumatic brain injury. Clin J Sport Med 2001;11:23–31. 10.1097/00042752-200101000-00005 11176142

[75] Guskiewicz KM, Marshall SW, Broglio SP, et al No evidence of impaired neurocognitive performance in collegiate soccer players. Am J Sports Med 2002;30:157–62. 11912081 10.1177/03635465020300020201

[76] Guskiewicz KM, McCrea M, Marshall SW, et al Cumulative effects associated with recurrent concussion in collegiate football players: the NCAA Concussion Study. JAMA 2003;290:2549–55. 10.1001/jama.290.19.2549 14625331

[77] Thornton AE, Cox DN, Whitfield K, et al Cumulative concussion exposure in rugby players: neurocognitive and symptomatic outcomes. J Clin Exp Neuropsychol 2008;30:398–409. 10.1080/13803390701443662 18938678

[78] Mioshi E, Dawson K, Mitchell J, et al The Addenbrooke's Cognitive Examination Revised (ACE-R): a brief cognitive test battery for dementia screening. Int J Geriatr Psychiatry 2006;21:1078–85. 10.1002/gps.1610 16977673

[79] Collie A, Maruff P, Makdissi M, et al CogSport: reliability and correlation with conventional cognitive tests used in postconcussion medical evaluations. Clin J Sport Med 2003;13:28–32. 10.1097/00042752-200301000-00006 12544161

[80] Hunt TN, Ferrara MS, Miller LS, et al The effect of effort on baseline neuropsychological test scores in high school football athletes. Arch Clin Neuropsychol 2007;22:615–21. 10.1016/j.acn.2007.04.005 17507199

[81] Schatz P Long-term test-retest reliability of baseline cognitive assessments using ImPACT. Am J Sports Med 2010;38:47–53. 10.1177/0363546509343805 19789333

[82] Solomon GS, Haase RF Biopsychosocial characteristics and neurocognitive test performance in National Football League players: an initial assessment. Arch Clin Neuropsychol 2008;23:563–77. 10.1016/j.acn.2008.05.008 18614333

[83] Warden DL, Gordon B, McAllister TW, et al; Neurobehavioral Guidelines Working Group. Guidelines for the pharmacologic treatment of neurobehavioral sequelae of traumatic brain injury. J Neurotrauma 2006;23:1468–501. 10.1089/neu.2006.23.1468 17020483

[84] Kim J, Whyte J, Patel S, et al Methylphenidate modulates sustained attention and cortical activation in survivors of traumatic brain injury: a perfusion fMRI study. Psychopharmacology (Berl) 2012;222:47–57. 10.1007/s00213-011-2622-8 22203319 PMC3369011

[85] Willmott C, Ponsford J Efficacy of methylphenidate in the rehabilitation of attention following traumatic brain injury: a randomised, crossover, double blind, placebo controlled inpatient trial. J Neurol Neurosurg Psychiatry 2009;80:552–7. 10.1136/jnnp.2008.159632 19060022

[86] Whyte J, Hart T, Vaccaro M, et al Effects of methylphenidate on attention deficits after traumatic brain injury: a multidimensional, randomized, controlled trial. Am J Phys Med Rehabil 2004;83:401–20. 10.1097/01.PHM.0000128789.75375.D3 15166683

[87] Pavlovskaya M, Hochstein S, Keren O, et al Methylphenidate effect on hemispheric attentional imbalance in patients with traumatic brain injury: a psychophysical study. Brain Inj 2007;21:489–97. 10.1080/02699050701311117 17522988

[88] Wheaton P, Mathias JL, Vink R Impact of pharmacological treatments on outcome in adult rodents after traumatic brain injury: a meta-analysis. J Psychopharmacol 2011;25:1581–99. 10.1177/0269881110388331 21300634

[89] Al-Adawi S, Calvanio R, Dorvlo A, et al The effect of methylphenidate on attention in acquired brain injury as recorded by useful field of view. J Appl Res 2005;5:61–72.

[90] Lee H, Kim SW, Kim JM, et al Comparing effects of methylphenidate, sertraline and placebo on neuropsychiatric sequelae in patients with traumatic brain injury. Hum Psychopharmacol 2005;20:97–104. 10.1002/hup.668 15641125

[91] Giacino JT, Whyte J, Bagiella E, et al Placebo-controlled trial of amantadine for severe traumatic brain injury. N Engl J Med 2012;366:819–26. 10.1056/NEJMoa1102609 22375973

[92] Meythaler JM, Brunner RC, Johnson A, et al Amantadine to improve neurorecovery in traumatic brain injury-associated diffuse axonal injury: a pilot double-blind randomized trial. J Head Trauma Rehabil 2002;17:300–13. 10.1097/00001199-200208000-00004 12105999

[93] Chung CS, Pollock A, Campbell T, et al Cognitive rehabilitation for executive dysfunction in adults with stroke or other adult non-progressive acquired brain damage. Cochrane Database Syst Rev 2013;4:CD008391 10.1002/14651858.CD008391.pub2 PMC6464714 23633354

[94] Krasny-Pacini A, Chevignard M, Evans J Goal Management Training for rehabilitation of executive functions: a systematic review of effectiveness in patients with acquired brain injury. Disabil Rehabil 2014;36:105–16. 10.3109/09638288.2013.777807 23597002

[95] Wilson BA, Emslie H, Quirk K, et al A randomized control trial to evaluate a paging system for people with traumatic brain injury. Brain Inj 2005;19:891–4. 10.1080/02699050400002363 16296571

[96] Cicerone KD, Dahlberg C, Malec JF, et al Evidence-based cognitive rehabilitation: updated review of the literature from 1998 through 2002. Arch Phys Med Rehabil 2005;86:1681–92. 10.1016/j.apmr.2005.03.024 16084827

[97] Chen SH, Thomas JD, Glueckauf RL, et al The effectiveness of computer-assisted cognitive rehabilitation for persons with traumatic brain injury. Brain Inj 1997;11:197–209. 10.1080/026990597123647 9058001

[98] De Luca R, Calabro RS, Gervasi G, et al Is computer-assisted training effective in improving rehabilitative outcomes after brain injury? A case-control hospital-based study. Disabil Health J 2014;7:356–60. 10.1016/j.dhjo.2014.04.003 24947578

[99] Fernandez E, Bringas ML, Salazar S, et al Clinical impact of RehaCom software for cognitive rehabilitation of patients with acquired brain injury. MEDICC Rev 2012;14:32–5. 10.1590/S1555-79602012000400007 23154316

[100] Fann JR, Uomoto JM, Katon WJ Cognitive improvement with treatment of depression following mild traumatic brain injury. Psychosomatics 2001;42:48–54. 10.1176/appi.psy.42.1.48 11161121

[101] Horsfield SA, Rosse RB, Tomasino V, et al Fluoxetine's effects on cognitive performance in patients with traumatic brain injury. Int J Psychiatry Med 2002;32:337–44. 10.2190/KQ48-XT0L-2H14-5UMV 12779183

[102] Bedard M, Felteau M, Marshall S, et al Mindfulness-based cognitive therapy reduces symptoms of depression in people with a traumatic brain injury: results from a randomized controlled trial. J Head Trauma Rehabil 2014;29:E13–22. 10.1097/HTR.0b013e3182a615a0 24052092

[103] Soo C, Tate R Psychological treatment for anxiety in people with traumatic brain injury. Cochrane Database Syst Rev 2007 (3):CD005239. 17636792 10.1002/14651858.CD005239.pub2 PMC12015660

[104] Fann JR, Uomoto JM, Katon WJ Sertraline in the treatment of major depression following mild traumatic brain injury. J Neuropsychiatry Clin Neurosci 2000;12:226–32. 10.1176/jnp.12.2.226 11001601

[105] Rapoport MJ, Chan F, Lanctot K, et al An open-label study of citalopram for major depression following traumatic brain injury. J Psychopharmacol 2008;22:860–4. 10.1177/0269881107083845 18208921

[106] Turner-Stokes L, Hassan N, Pierce K, et al Managing depression in brain injury rehabilitation: the use of an integrated care pathway and preliminary report of response to sertraline. Clin Rehabil 2002;16:261–8. 10.1191/0269215502cr489oa 12017513

[107] Lucas S Headache management in concussion and mild traumatic brain injury. PMR 2011;3 (10 Suppl 2):S406–12. 10.1016/j.pmrj.2011.07.016 22035683

[108] Hecht JS Occipital nerve blocks in postconcussive headaches: a retrospective review and report of ten patients. J Head Trauma Rehabil 2004;19:58–71. 10.1097/00001199-200401000-00006 14732831

[109] Chaibi A, Russell MB Manual therapies for cervicogenic headache: a systematic review. J Headache Pain 2012;13:351–9. 10.1007/s10194-012-0436-7 22460941 PMC3381059

[110] Inan N, Ceyhan A, Inan L, et al C2/C3 nerve blocks and greater occipital nerve block in cervicogenic headache treatment. Funct Neurol 2001;16:239–43. 11769869

[111] Kaski D, Bronstein AM Epley and beyond: an update on treating positional vertigo. Pract Neurol 2014;14:210–21. 10.1136/practneurol-2013-000690 24570475

[112] Alsalaheen BA, Mucha A, Morris LO, et al Vestibular rehabilitation for dizziness and balance disorders after concussion. J Neurol Phys Ther 2010;34:87–93. 10.1097/NPT.0b013e3181dde568 20588094

[113] Ouellet MC, Morin CM Efficacy of cognitive-behavioral therapy for insomnia associated with traumatic brain injury: a single-case experimental design. Arch Phys Med Rehabil 2007;88:1581–92. 10.1016/j.apmr.2007.09.006 18047872

[114] Larson EB, Zollman FS The effect of sleep medications on cognitive recovery from traumatic brain injury. J Head Trauma Rehabil 2010;25:61–7. 10.1097/HTR.0b013e3181c1d1e1 20051895

[115] Kaiser PR, Valko PO, Werth E, et al Modafinil ameliorates excessive daytime sleepiness after traumatic brain injury. Neurology 2010;75:1780–5. 10.1212/WNL.0b013e3181fd62a2 21079179

[116] Klose M, Feldt-Rasmussen U Does the type and severity of brain injury predict hypothalamo-pituitary dysfunction? Does post-traumatic hypopituitarism predict worse outcome? Pituitary 2008;11:255–61. 10.1007/s11102-008-0102-x 18404391

[117] Sinclair KL, Ponsford JL, Taffe J, et al Randomized controlled trial of light therapy for fatigue following traumatic brain injury. Neurorehabil Neural Repair 2014;28:303–13. 10.1177/1545968313508472 24213962

[118] Jankowski LW, Sullivan SJ Aerobic and neuromuscular training: effect on the capacity, efficiency, and fatigability of patients with traumatic brain injuries. Arch Phys Med Rehabil 1990;71:500–4. 2350220

[119] Sullivan SJ, Richer E, Laurent F The role of and possibilities for physical conditioning programmes in the rehabilitation of traumatically brain-injured persons. Brain Inj 1990;4:407–14. 10.3109/02699059009026194 2252972

[120] Fleminger S Mental Health is Central to Good Neurorehabilitation after TBI. Brain Impairment 2013;14.01:2–4. 10.1017/BrImp.2013.14

[121] Fazel S, Wolf A, Pillas D, et al Suicide, fatal injuries, and other causes of premature mortality in patients with traumatic brain injury: a 41-year Swedish population study. JAMA Psychiatry 2014;71:326–33. 10.1001/jamapsychiatry.2013.3935 24430827 PMC4058552

[122] Whitnall L, McMillan TM, Murray GD, et al Disability in young people and adults after head injury: 5–7 year follow up of a prospective cohort study. J Neurol Neurosurg Psychiatry 2006;77:640–5. 10.1136/jnnp.2005.078246 16614025 PMC2117429

[123] Gould KR, Ponsford JL, Johnston L, et al Relationship between psychiatric disorders and 1-year psychosocial outcome following traumatic brain injury. J Head Trauma Rehabil 2011;26:79–89. 10.1097/HTR.0b013e3182036799 21209565

[124] Gould KR, Ponsford JL, Johnston L, et al The nature, frequency and course of psychiatric disorders in the first year after traumatic brain injury: a prospective study. Psychol Med 2011;41:2099–109. 10.1017/S003329171100033X 21477420

[125] Jorge RE, Robinson RG, Moser D, et al Major depression following traumatic brain injury. Arch Gen Psychiatry 2004;61:42–50. 10.1001/archpsyc.61.1.42 14706943

[126] Silver JM, McAllister TW, Arciniegas DB Depression and cognitive complaints following mild traumatic brain injury. Am J Psychiatry 2009;166:653–61. 10.1176/appi.ajp.2009.08111676 19487401

[127] Bryant RA, Moulds M, Guthrie R, et al Treating acute stress disorder following mild traumatic brain injury. Am J Psychiatry 2003;160:585–7. 10.1176/appi.ajp.160.3.585 12611847

[128] Tiersky LA, Anselmi V, Johnston MV, et al A trial of neuropsychologic rehabilitation in mild-spectrum traumatic brain injury. Arch Phys Med Rehabil 2005;86:1565–74. 10.1016/j.apmr.2005.03.013 16084809

[129] Hoffman JM, Lucas S, Dikmen S, et al Natural history of headache after traumatic brain injury. J Neurotrauma 2011;28:1719–25. 10.1089/neu.2011.1914 21732765 PMC3172878

[130] Lucas S, Hoffman JM, Bell KR, et al A prospective study of prevalence and characterization of headache following mild traumatic brain injury. Cephalalgia 2014;34:93–102. 10.1177/0333102413499645 23921798

[131] Walker WC, Marwitz JH, Wilk AR, et al Prediction of headache severity (density and functional impact) after traumatic brain injury: a longitudinal multicenter study. Cephalalgia 2013;33:998–1008. 10.1177/0333102413482197 23575819

[132] Packard RC, Ham LP Pathogenesis of posttraumatic headache and migraine: a common headache pathway? Headache 1997;37:142–52. 10.1046/j.1526-4610.1997.3703142.x 9100398

[133] Lucas S, Hoffman JM, Bell KR, et al Characterization of headache after traumatic brain injury. Cephalalgia 2012;32:600–6. 10.1177/0333102412445224 22623761

[134] Erickson JC Treatment outcomes of chronic post-traumatic headaches after mild head trauma in US soldiers: an observational study. Headache 2011;51:932–44. 10.1111/j.1526-4610.2011.01909.x 21592097

[135] Kisilevski V, Podoshin L, Ben-David J, et al Results of otovestibular tests in mild head injuries. Int Tinnitus J 2001;7:118–21. 14689650

[136] Berman JM, Fredrickson JM Vertigo after head injury—a five year follow-up. J Otolaryngol 1978;7:237–45. 151151

[137] Maskell F, Chiarelli P, Isles R Dizziness after traumatic brain injury: overview and measurement in the clinical setting. Brain Inj 2006;20:293–305. 10.1080/02699050500488041 16537271

[138] Bronstein AM, Lempert T, Seemungal BM Chronic dizziness: a practical approach. Pract Neurol 2010;10:129–39. 10.1136/jnnp.2010.211607 20498184

[139] Ahn SK, Jeon SY, Kim JP, et al Clinical characteristics and treatment of benign paroxysmal positional vertigo after traumatic brain injury. J Trauma 2011;70:442–6. 10.1097/TA.0b013e3181d0c3d9 20489667

[140] Liu H Presentation and outcome of post-traumatic benign paroxysmal positional vertigo. Acta Otolaryngol 2012;132:803–6. 10.3109/00016489.2012.657359 22404210

[141] Yardley L Prediction of handicap and emotional distress in patients with recurrent vertigo: symptoms, coping strategies, control beliefs and reciprocal causation. Soc Sci Med 1994;39:573–81. 10.1016/0277-9536(94)90100-7 7973857

[142] Johns M, Hocking B Daytime sleepiness and sleep habits of Australian workers. Sleep 1997;20:844–9. 9415943 10.1093/sleep/20.10.844

[143] Buysse DJ, Reynolds CF III, Monk TH, et al The Pittsburgh Sleep Quality Index: a new instrument for psychiatric practice and research. Psychiatry Res 1989;28: 193–213. 10.1016/0165-1781(89)90047-4 2748771

[144] Mathias JL, Alvaro PK Prevalence of sleep disturbances, disorders, and problems following traumatic brain injury: a meta-analysis. Sleep Med 2012;13:898–905. 10.1016/j.sleep.2012.04.006 22705246

[145] Shekleton JA, Parcell DL, Redman JR, et al Sleep disturbance and melatonin levels following traumatic brain injury. Neurology 2010;74:1732–8. 10.1212/WNL.0b013e3181e0438b 20498441 PMC3462582

